# Circadian Governance of Cardiac Growth

**DOI:** 10.3390/cells11091494

**Published:** 2022-04-29

**Authors:** Mary N. Latimer, Martin E. Young

**Affiliations:** Division of Cardiovascular Disease, Department of Medicine, University of Alabama at Birmingham, Birmingham, AL 35294, USA; marylatimer@uab.edu

**Keywords:** chronobiology, growth, heart, metabolism, signaling

## Abstract

The cardiomyocyte circadian clock temporally governs fundamental cellular processes, leading to 24-h rhythms in cardiac properties (such as electrophysiology and contractility). The importance of this cell-autonomous clock is underscored by reports that the disruption of the mechanism leads to adverse cardiac remodeling and heart failure. In healthy non-stressed mice, the cardiomyocyte circadian clock modestly augments both cardiac protein synthesis (~14%) and mass (~11%) at the awake-to-sleep transition (relative to their lowest values in the middle of the awake period). However, the increased capacity for cardiac growth at the awake-to-sleep transition exacerbates the responsiveness of the heart to pro-hypertrophic stimuli/stresses (e.g., adrenergic stimulation, nutrients) at this time. The cardiomyocyte circadian clock orchestrates time-of-day-dependent rhythms in cardiac growth through numerous mechanisms. Both ribosomal RNA (e.g., 28S) and the PI3K/AKT/mTOR/S6 signaling axis are circadian regulated, peaking at the awake-to-sleep transition in the heart. Conversely, the negative regulators of translation (including PER2, AMPK, and the integrated stress response) are elevated in the middle of the awake period in a coordinated fashion. We speculate that persistent circadian governance of cardiac growth during non-dipping/nocturnal hypertension, sleep apnea, and/or shift work may exacerbate left ventricular hypertrophy and cardiac disease development, highlighting a need for the advancement of chronotherapeutic interventions.

## 1. Introduction

Biological processes are influenced by the time of day to varying extents. In mammals, daily fluctuations are readily apparent at behavioral (e.g., sleep, physical activity, food intake), organ/system (e.g., cognitive, endocrine, cardiovascular functions), and cellular (e.g., transcription, translation, post-translational events) levels [1,2]. In the case of the heart, 24-h oscillations have been reported for several contractility parameters (e.g., heart rate, diastolic function) in humans and animal models, which have classically been attributed to changes in the neurohumoral milieu (often secondary to behaviors) [3,4,5,6,7]. However, it has become increasingly clear that the heart exhibits distinct molecular and biochemical signatures depending on the time of day, such that the intrinsic properties of the myocardium differ markedly between day and night [8,9,10]. Many daily changes in the intrinsic properties of the myocardium cannot be explained simply by fluctuations in the neurohumoral environment, but instead appear to be driven in large part by a cell-autonomous timekeeping mechanism known as the circadian clock [8,9,10]. Circadian governance orchestrates a plethora of fundamental cardiac processes, ensuring that they occur in a temporally appropriate manner. These processes range from transcription, translation, and post-translational modifications, to electrophysiology, signaling, and metabolism (including the synthesis and degradation of cellular constituents) [1,2]. This review article will outline the current knowledge regarding circadian regulation of cardiac growth, with a particular focus on hypertrophy (as opposed to hyperplasia) and protein synthesis. Moreover, the consequences of these daily rhythms, in terms of the responsiveness of the myocardium to pro-hypertrophic stimuli, will be discussed, as will the potential translational implications.

## 2. The Awake-to-Sleep Phase Transition Is a Period of Pro-Hypertrophic Growth

The temporal orchestration of cardiac metabolism (defined collectively as the processes involved in synthesis and/or degradation of cellular constituents) is closely aligned with predictable daily cycles in prominent behaviors (such as sleep-wake and fasting-feeding cycles) [11,12]. Here, we provide a brief overview of the circadian regulation of cardiac metabolism, based primarily on in vivo and ex vivo tracer studies in rodents. Oxidative metabolism (particularly glucose oxidation) is augmented at the beginning of the active period, coincident with increased energetic demand at this time (secondary to enhanced physical activity and cardiac contractility) [13,14,15]. During the latter half of the active period, nutrients that exceed energetic requirements are utilized for the synthesis of intracellular fuel stores (i.e., glycogen and triglyceride) [14,16]; during the subsequent sleep-associated fasting period, these stores provide carbon for both catabolic (i.e., ATP generation) and anabolic (e.g., protein synthesis) reactions. In the latter case, myocardial protein synthesis is elevated at the awake-to-sleep transition, followed by the activation of processes involved in the degradation of damaged proteins (e.g., autophagy) approximately 4 h later [17,18]. In doing so, the early portion of the sleep period can be considered a time of cellular maintenance/repair, likely ensuring optimal cardiac function upon awakening. Given that cellular constituent turnover plays a critical role during both the physiologic and pathologic states, the awake-to-sleep transition can be considered an important time period for myocardial health.

During a normal 24-h period, the consequences related to the temporal governance of cellular constituent turnover might not be readily apparent. Despite rates of protein synthesis being highest at the end of the active period, cardiac mass fluctuates only modestly over the course of the day, requiring a relatively large number of observations to detect the ~11% higher biventricular weight of the murine heart at the awake-to-sleep transition (versus the middle of the active period) [19]. The modest nature of oscillations in this gravimetric parameter is likely secondary to multiple factors. First, the peak in protein synthesis occurs just 4 h prior to the peak in autophagy, thereby minimizing net protein accumulation [17,18]. Second, the window of increased protein synthesis is relatively brief and is preceded 6 h earlier by the lowest daily rates of myocardial protein synthesis (which occur in the middle of the active period) [17]. Finally, the trough-to-peak differences in myocardial protein synthesis are relatively modest under non-stressed conditions, representing an ~14% increase at the awake-to-sleep transition (relative to the trough occurring 6 h earlier) [17]. The aforementioned temporal sequence of events, combined with the transient nature and magnitude of the protein synthesis peak, is consistent with a role for the maintenance of cellular integrity (as opposed to daily growth of the myocardium). However, the potential impact of this circadian governance becomes more evident in the presence of a stimulus/stress. The growth of the myocardium in response to physiologic stimuli (e.g., exercise) or pathologic stresses (e.g., pressure/volume overload) requires the activation of myocardial remodeling programs; this involves not only the synthesis of new protein, but also the removal of older/damaged cellular constituents [20]. As highlighted above, these processes are primed at the awake-to-sleep transition, such that remodeling of the myocardium is exacerbated in the presence of stimuli/stresses at this time-of-day. Two examples include isoproterenol and branched chain amino acids (BCAAs). Challenging mice with isoproterenol specifically at the awake-to-sleep transition on a daily basis (for a 1-week period) leads to a significant increase in cardiac mass and the induction of molecular markers of hypertrophy (e.g., anf); in contrast, the same stimulus at the sleep-to-awake transition is without effect [21]. Similarly, feeding mice a high BCAA meal at the end of the awake period led to a rapid (within 4 h) increase in cardiac mass, cardiomyocyte size, and myocardial protein synthesis; the same meal at the beginning of the active period had no effect on these parameters [22]. In the latter study, hypertrophic growth was transient in nature; these data suggested that the 70% increase in cardiomyocyte size at the end of the 4 h meal returned to a pre-challenge size within 6 h, consistent with increased rates of autophagy as the sleep phase progresses [22]. It’s also noteworthy that Gibb et al. reported that exercising FVB/NJ male mice at the awake-to-sleep transition for 4 weeks resulted in increased heart weight to tibia length ratios (relative to mice exercised at the sleep-to-awake transition) [23]. Collectively, these studies highlight that the heart is primed for growth at the awake-to-sleep phase transition.

## 3. Contribution of Extrinsic versus Intrinsic Factors

Numerous extracellular stimuli and intracellular mechanisms that regulate cardiac growth have been described. These can be divided into the following two broad categories: factors that are extrinsic or intrinsic to the heart. Extrinsic factors known to influence cardiac growth range from blood pressure, diet, and physical activity to neurohumoral factors such as epinephrine/norepinephrine, cortisol, nutrients, insulin, thyroid hormone, and growth hormone [24,25,26,27,28,29,30]. Many of these parameters fluctuate over the course of the day, leading to the possibility that they contribute towards 24-h rhythms in cardiac growth [31]. However, the timing of their peak does not always align with that of myocardial protein synthesis. For example, in the rodent, blood pressure, food intake, physical activity, adrenergic stimulation, cortisol, and insulin all peak within the first half of the active period (a time at which myocardial protein synthesis and growth capacity are low) [5,18,32,33]. However, it is noteworthy that some of these parameters can exhibit a secondary peak at the end of the active period; these include blood pressure, physical activity, and food intake [18,32]. Growth hormone is typically secreted in a pulsatile fashion, with a higher amplitude during the sleep period [34,35]. In rodents, peak growth hormone levels tend to be observed towards the awake-to-sleep transition [33]. In addition, several key components of the renin-angiotensin-aldosterone system (RAAS) are elevated at the awake-to-sleep transition in the rodent heart (particularly during hypertension), including renin, angiotensin converting enzyme, and angiotensin receptors 1a and 2 [36]. Collectively, these observations suggest that a number of endocrine (e.g., growth hormone) and paracrine (e.g., RAAS) factors, in addition to sheer stress, have the potential to contribute towards increased cardiac growth at the awake-to-sleep transition.

**Figure 1:**
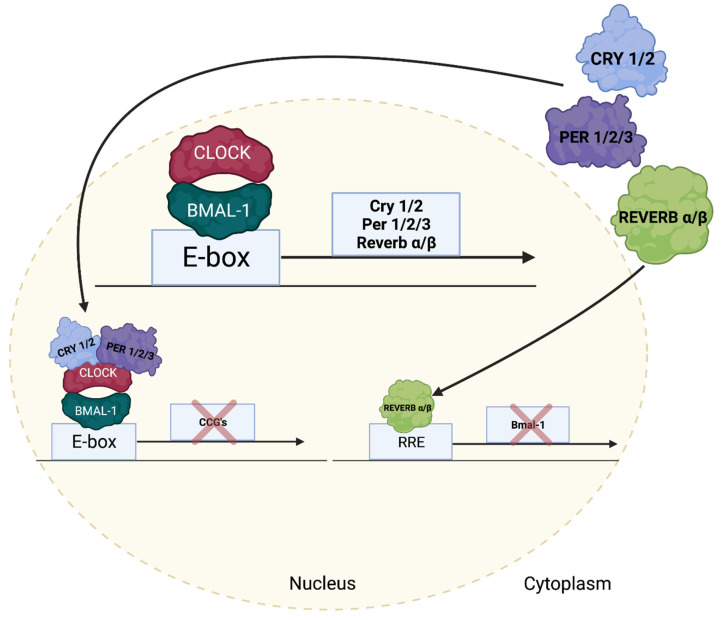
The Mammalian Circadian Clock Mechanism. The circadian clock is a transcriptional and post-translational-based mechanism composed of a series of positive and negative feedback loops, with a free-running period of ~24 h. Core clock components, and their interrelationship, are illustrated.

It is clear that myocardial responsiveness to pro-hypertrophic stimuli fluctuates over the course of the day. One possible explanation is that multiple extracardiac factors interact, such that one impacts the action of another. For example, it is well established that nutrients, cortisol, thyroid hormone, and physical activity influence the insulin sensitivity of various tissues [37]. In addition, the sensitivity of cells to extracellular stimuli can also be modulated over the course of the day by an intrinsic timekeeping mechanism known as the circadian clock. Indeed, a primary function of this mechanism is to allow cells to anticipate changes in the environment before they occur, ensuring that the responsiveness of the cell is temporally appropriate. Circadian clocks are self-sustained cell-autonomous transcriptional/translation feedback loops with a free-running period of approximately 24 h that have been characterized in virtually all mammalian cells (including cardiomyocytes) [1,2,38]. At the heart of the mechanism are two transcription factors, CLOCK and BMAL1 (Figure 1) [39,40]. Upon heterodimerization, the CLOCK/BMAL1 heterodimer binds to E-boxes in target genes, leading to induction; these target genes include negative feedback loop components, such as period (PER1/2/3), cryptochrome (CRY1/2), and REV-ERB (REV-ERBα/β) isoforms [41,42,43]. For example, following accumulation at the protein level, PER/CRY heterodimers translocate to the nucleus and inhibit the transcriptional activity of the CLOCK/BMAL1 heterodimer [44,45]. In the case of REV-ERBα/β, these transcription factors bind to the promoter of the *Bmal1* gene, leading to transcriptional repression [43]. As alluded to, all clock components detailed thus far are transcriptional modulators and therefore have the capability to influence a host of target genes that are not considered integral to the core clock mechanism; these are termed clock output or clock-controlled genes. It has been estimated that cell autonomous clocks modulate 3-16% of a tissue’s transcriptome; many of these genes encode for proteins involved in transcription, translation, electrophysiology, signaling, and metabolism [46]. These processes are critical for maintenance of normal cardiac function.

Considerable evidence has accumulated in support of the concept that the cardiomyocyte circadian clock plays a key role in augmenting cardiac growth at the awake-to-sleep transition. Much of this evidence has been acquired using murine models of genetic disruption of this timekeeping mechanism. To date, the two most common models have targeted the central CLOCK/BMAL1 heterodimer; namely cardiomyocyte-specific CLOCK mutant (CCM; targeted expression of a dominant negative CLOCK mutant protein in cardiomyocytes) and cardiomyocyte-specific BMAL1 knockout (CBK) mice [15,47]. Transcriptomic studies in CCM and CBK mouse hearts indicated that genetic disruption to the central CLOCK/BMAL1 heterodimer led to the temporal suspension of the cardiac transcriptome at the awake-to-sleep phase transition [15,47]. More specifically, genes that oscillate over the course of the day in control (i.e., wild-type) hearts, remained at constant levels in the CCM and CBK hearts (with the relative expression corresponding to levels in wild-type hearts at the awake-to-sleep transition). Consistent with the latter being a pro-hypertrophic time-of-day, both CCM and CBK mice exhibit increased cardiac mass and cardiomyocyte size at a relatively young age [15,47,48]. Similarly, cardiac protein synthesis rates do not oscillate in CBK hearts, but instead remain ‘locked’ at the high level observed in wild-type hearts at the awake-to-sleep phase transition (protein synthesis rates have not been reported in CCM hearts) [17]. It is equally important to note that time-of-day-dependent oscillations in responsiveness of the heart to distinct pro-hypertrophic stimuli (i.e., isoproterenol and BCAAs) are abolished in CCM and CBK hearts [21,22]. These observations indicate that the cardiomyocyte circadian clock promotes cardiac growth during the awake-to-sleep transition.

## 4. Lessons Learned from Extra-Cardiac Tissues

The concept that cellular growth occurs in a temporal fashion is not entirely novel. Several examples exist, indicating that growth occurs during distinct time periods. A textbook example is the cell cycle. In proliferating cells, protein synthesis is augmented during both the G1 and G2 phases; the G2 phase is considered a fidelity period, during which the cellular constituent integrity is checked prior to the upcoming mitosis [49]. Interestingly, it has been postulated that an evolutionarily conserved role of circadian clocks is the temporal gating of distinct cell cycle stages, to ensure that distinct processes occur at an appropriate time-of-day. Circadian governance is thought to confine the S phase (i.e., DNA synthesis) to the dark period, while DNA repair occurs during the light phase; in doing so, the transmission of UV-induced DNA damage to daughter cells is minimized [50,51]. When the circadian clock mechanism is disrupted, temporal control of the cell cycle becomes impaired, leading to increased tumorigenesis [50,52]. Consistent with this concept, night shift workers have higher incidence of specific forms of cancer [53,54]. Instead of focusing on hyperplasia, this subsection outlines the insight gained from studies investigating circadian regulation of hypertrophic growth in extra-cardiac tissues.

Numerous studies have investigated circadian governance of translation and growth in the liver. This stems in part from two seminal observations. First, the comparison of 24-h fluctuations in the hepatic transcriptome and proteome revealed that only ~50% of the proteins exhibiting circadian rhythms could be explained by alterations in corresponding mRNA species, suggestive of circadian regulation at the post-transcriptional level [55]. Second, liver weight changes over the course of the day, being ~34% larger at the awake-to-sleep phase transition; these gravimetric changes appear to be primarily due to fluctuations in protein synthesis, as opposed to glycogen and/or water content, and are mirrored by oscillations in hepatocyte size [56]. Collectively, these data suggest that circadian regulation of translation and/or protein degradation leads to time-of-day-dependent fluctuations in hepatic growth. Various mechanisms have been proposed for mediating the temporal regulation of hepatic growth, many of which have direct established links to the circadian clock. For example, Lipton et al. reported that BMAL1, in addition to being a transcription factor, is an integral component of the translational machinery [57]. More specifically, upon phosphorylation by S6K1, BMAL1 forms a complex with eIF3B, eIF4F, and PABP in the cytosol, leading to the facilitation of protein synthesis. Consistent with this, 24-h oscillations in protein synthesis were observed in both MEFs (in vitro) and livers (in vivo); for the in vivo studies, protein synthesis rates were augmented at the awake-to-sleep phase transition (a time at which BMAL1 and p-S6K1 levels are both elevated). The deletion of *Bmal1* abolished these oscillations [57]. Conversely, daily rhythms may be magnified by selectively attenuating protein synthesis at the time of the trough (i.e., the sleep-to-awake transition), by the negative circadian clock component PER2. More specifically, Wu et al. reported that PER2 binds to mTORC1, leading to the suppression of hepatic protein synthesis (and promotion of autophagy) [58]. It is noteworthy that S6K1 activity is dependent on mTORC1, such that high levels of PER2 at the sleep-to-wake transition is predicted to attenuate BMAL1 association with the transcriptional machinery through the prevention of S6K1 activation. Thus, independent of their classic role as transcriptional modulators, positive (BMAL1) and negative (PER2) circadian clock components directly contribute towards the temporal control of hepatic protein synthesis.

Circadian clocks influence protein synthesis through a number of additional mechanisms. This includes the regulation of rRNA turnover. Sinturel et al. reported that although transcription of rRNA is constant over the course of the day, rRNA degradation exhibits a 24-h oscillation [56]. More specifically, rRNA polyadenylation and subsequent degradation in the liver is antiphase to ribosome assembly and protein synthesis [56]. In addition to rRNA, circadian regulation via a tRNA-dependent mechanism has been suggested. When amino acid availability decreases, the proportion of charged tRNA in the cell may decrease, triggering the integrated stress response (ISR), via activation of GCN2 and subsequent phosphorylation of eIF2α [59,60]. The latter phosphorylation event inhibits the formation of the eIF2-GTP-methionyl-tRNA complex that is required for translation initiation of the majority of mRNA-encoded proteins [61]. Studies in Neurospora reveal that both uncharged tRNAs and eIF2α phosphorylation exhibit time-of-day-dependent oscillations, and that p-eIF2α rhythms are abolished following genetic disruption of either the circadian clock or GCN2 [62]. Similarly, p-eIF2α oscillates in the suprachiasmatic nucleus and liver, which is severely attenuated in GCN2 knockout mice; in the liver, peak p-eIF2α levels were observed during the first 4 h of the active period, consistent with low rates of protein synthesis at this time [63].

It is noteworthy that cell-autonomous clocks have the potential to influence protein synthesis and cellular growth through various transcriptional and post-translational mechanisms. Through the use of unbiased omics-based approaches, biological processes such as translation and cellular signaling have been identified as circadian regulated in numerous tissues [46]. A comprehensive study by Zhang et al. exemplifies this; the investigators compared the transcriptomes of 12 different mouse tissues through the use of RNAseq, revealing extensive and divergent circadian regulation of gene expression [46]. When comparing between tissues, the overlap of circadian governance was observed for multiple signaling components known to impact protein synthesis (including several components of the IGF1/PI3K/AKT/mTOR signaling axis) [46]. This is underscored by hepatic phosphoproteome studies, which uncovered that ~25% of phosphoproteins in the liver oscillate over a 24-h period; of these, AKT1, mTOR, and P70S6K were found to peak towards the middle of the active period [64]. This study also identified AMPK as being circadian regulated [64]. Given that AMPK inhibits protein synthesis (through phosphorylation of TSC2 and subsequent suppression of mTORC1 activity), a gradual increase in hepatic AMPK levels as the sleep phase progresses may play a role in the suppression of hepatocyte growth close to awakening [65,66].

**Figure 2:**
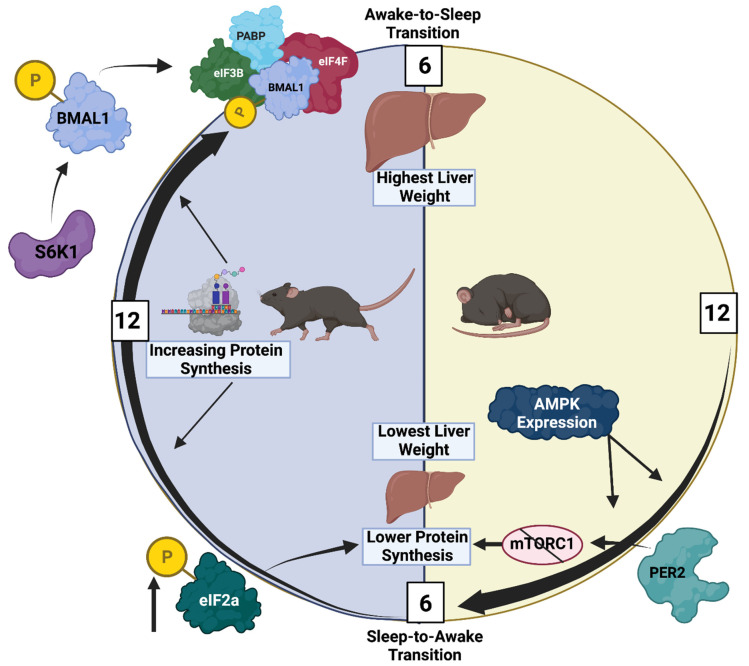
Circadian Regulation of Hepatic Protein Synthesis and Growth. As illustrated in the figure, hepatic mass reaches a peak at the awake-to-sleep transition, secondary to high protein synthesis rates during the awake period. The latter is promoted by a steady increase in S6K1 activity and BMAL1 phosphorylation, leading to complex formation with eIF3B, eIF4F, and PABP, and subsequent translation initiation. Similarly, rRNA levels increase during the awake period, facilitating ribosome assembly and protein synthesis. Conversely, the increased expression of AMPK and activation of the integrated stress response as the sleep phase progresses attenuates protein synthesis at this time.

In summary (Figure 2), significant evidence suggests that the hepatocyte circadian clock temporally orchestrates multiple mechanisms, culminating in the promotion of protein synthesis and cellular growth during the active period, followed by the repression of these processes during the sleep period.

## 5. Regulation of Cardiac Growth Pathways by the Cardiomyocyte Circadian Clock

Using knowledge gained from the liver as a starting point, hypothetical mechanisms by which the cardiomyocyte circadian clock governs temporal control of cardiac growth can be considered. Similar to the liver, *Bmal1* and *Per2* mRNA levels exhibit striking oscillations in the heart [10,38,67]. Elevated levels of *Bmal1* mRNA at the awake-to-sleep transition coincide with augmented protein synthesis [47]. However, time-of-day-dependent fluctuations in cardiac BMAL1 protein levels are negligible, and genetic deletion of BMAL1 in cardiomyocytes results in enhanced cardiac protein synthesis (opposite to prediction based on the role of BMAL1 as an integral component of the translation machinery) [14,17]. *Per2* mRNA rhythms peak approximately 6 h before the time of lowest cardiac protein synthesis; this phase difference might be explained by a delay in PER2 protein accumulation, which peaks in the middle of the awake period (in alignment with decreased protein synthesis) [14,17,47]. Interestingly, cardiomyocyte-specific disruption of the circadian clock has no impact on diurnal rhythms in cardiac *Per2* mRNA levels (an observation that has been reported in additional murine models of cell-type specific clock disruption, consistent with the responsiveness of the Per2 gene to neurohumoral factors); this is despite the abolition of time-of-day-dependent rhythms in protein synthesis following clock disruption [17,47,68]. Collectively, these observations suggest that although fluctuations in PER2 may inhibit cardiac protein synthesis during the middle of the awake period, the contribution of this core clock component towards augmented protein synthesis following circadian disruption is less clear.

Fluctuations in ribosomal RNA levels have the potential to contribute towards time-of-day-dependent rhythms in cardiac protein synthesis. Specifically, 28S rRNA levels exhibit an approximate 2-fold oscillation in the murine heart, peaking at the awake-to-sleep transition [22]. Somewhat surprisingly, these rhythms persist following the disruption of the cardiomyocyte circadian clock [22]. Although cardiac 18S rRNA levels do not fluctuate over a 24-h period, this rRNA is chronically elevated in CBK hearts (consistent with increased protein synthesis) [22]. The circadian regulation of the ISR (i.e., tRNA/GCN2/eIF2α axis) has not been reported in the heart to date. To gain insight into the potential role of this pathway, we have recently investigated cardiac p-eIF2α levels, observing ~2-fold higher levels in the middle of the active period (time of lowest protein synthesis) versus the awake-to-sleep phase transition (time of highest protein synthesis); these diurnal variations are lost in CBK hearts (for which p-eIF2α levels are chronically low, consistent with high rates of protein synthesis; unpublished observations). The daily fluctuations in rRNA and the ISR are therefore consistent with diurnal variations in cardiac protein synthesis, while the impairment of the ISR following disruption of the cardiomyocyte circadian clock potentially contributes towards augmented cardiac growth.

**Figure 3:**
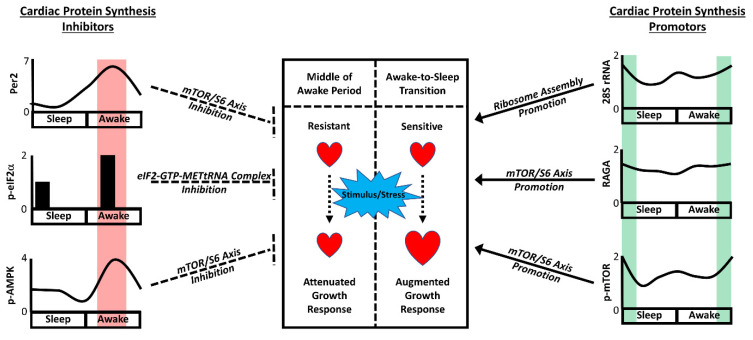
Circadian Orchestration of Anti- and Pro-Growth Mechanisms in the Heart. As highlighted in the figure, the heart is primed for growth at the awake-to-sleep transition, through coordinated alignment of mechanisms promoting protein synthesis at this time (green shaded area). Conversely, the cardiac growth response is greatly hindered in the middle of the awake period, due to elevated PER2, p-eIF2α, and AMPK at this time (red shaded area).

The circadian governance of cellular signaling pathways known to influence cardiac protein synthesis and cellular growth have been investigated. This includes the PI3K/AKT/mTOR/S6 signaling axis. Numerous studies indicate that the activity status of this axis increases during the awake period, peaking towards the awake-to-sleep phase transition [17,22,33]. Moreover, the peak activity status persists during fasting, suggesting that these rhythms are not secondary to feeding/fasting cycles [18]. These rhythms are lost in CBK hearts in a manner that leads to chronic activation of this signaling axis [17,22]. The mTOR inhibitor rapamycin has been utilized in multiple independent studies for establishing causal relationships. More specifically, rapamycin not only normalizes protein synthesis and cardiac mass in CBK hearts, but also abolishes the ability of BCAAs to augment cardiac growth at the awake-to-sleep transition [17,22]. Several mechanistic explanations for clock control of this axis exist (beyond the aforementioned binding of PER2 to mTORC1). First, the regulatory subunit of PI3K (p85α) is directly regulated at a transcriptional level by the CLOCK/BMAL1 heterodimer in cardiomyocytes [47]. Cardiac p85α levels are at their highest in the heart at the awake-to-sleep transition, a time at which insulin-mediated Akt activation is increased [17]. Second, we have recently reported that CBK hearts exhibit an increased sensitivity to growth hormone, associated with increased cardiac *Igf1* mRNA levels; when one allele of the Igf1 gene is deleted in CBK hearts, cardiac mass and cardiomyocyte size partially normalize, suggesting that excessive IGF1 signaling (principally acting through the AKT/mTOR/S6 signaling axis) contributes to augmented protein synthesis and cardiac growth [69]. Third, mTOR activity is augmented by numerous amino acids (including BCAAs); mass spectrometry revealed that cardiac amino acid levels are higher at the awake-to-sleep transition, whereas levels are ~15% higher in CBK hearts (independent of the time of day) [22]. Moreover, the ability of amino acids to activate mTOR is dependent on numerous ancillary proteins, acting in either positive (e.g., RAGA/D) or negative (e.g., DEPTOR) manners [70,71]. Cardiac RAGA and RAGD levels are higher, while DEPTOR is lower, at the awake-to-sleep phase transition (consistent with an increased ability of BCAA to active protein synthesis at this time); these diurnal variations are abolished in CBK hearts, which exhibit chronically high RAGA and RAGD levels and lower DEPTOR levels [22]. Finally, AMPK phosphorylation status and activity are both increased in the heart in the middle of the active period (which would be predicted to augment TSC2, attenuate mTORC1, and ultimately decrease protein synthesis); in contrast, cardiac AMPK phosphorylation/activity is low at the awake-to-sleep transition [16]. The diurnal variations in AMPK are governed by the cardiomyocyte circadian clock; the genetic disruption of this mechanism leads to chronically low AMPK activity [16]. Taken together, these studies illustrate that the cardiomyocyte circadian clock temporally orchestrates a number of mechanisms (several of which converge on mTOR) that mediate time-of-day-dependent rhythms in cardiac protein synthesis and growth (Figure 3).

## 6. Translational Implications

The appreciation of circadian control of cardiac growth (and remodeling) has implications at various levels, from bench to bedside. In terms of experimental considerations, the time of day at which acute challenges are performed, assessments are made, and samples are collected, has the potential to dramatically influence outcomes (as exemplified by the aforementioned animal-based isoproterenol and BCAA challenge studies). In this subsection, we focus more on potential translational implications. Given that the heart is primed for growth and remodeling at the awake-to-sleep phase transition, avoiding pro-hypertrophic stimuli at this time might be considered beneficial, especially in at-risk subjects (e.g., heart disease patients). Conversely, in healthy individuals, the possibility exists that the cardiovascular benefits of exercise might be augmented when exercise sessions and nutrient intake are coupled in a temporally appropriate manner. Epidemiologic, clinical, and basic/translational studies addressing questions such as these are discussed herein.

Several clinical states and lifestyle behaviors result in the heart being challenged with pro-hypertrophic stimuli during the ‘usual’ sleep period. These include non-dipping/nocturnal hypertension, sleep apnea, and night-shift work. In healthy individuals, blood pressure decreases during sleep [3]. In contrast, blood pressure is abnormally high during sleep in individuals with non-dipping or nocturnal hypertension [72]. Verdecchia and colleagues reported an inverse correlation between the level of blood pressure reduction at night and left ventricular mass, such that higher blood pressure during the sleep period was associated with greater cardiac hypertrophy [73]. In addition to elevating cardiac afterload during sleep, sleep apnea increases sympathetic tone, a pro-hypertrophic stimulus that typically declines during the sleep phase in healthy individuals [74]. Sleep apnea markedly increases risk of heart failure, in part due to adverse remodeling of the myocardium (including hypertrophic growth) [74]. Similarly, night shift work increases risk of numerous cardiovascular diseases (particularly adverse ischemic events) [75,76]. One study in shift working airline crews reported increased incidence of left ventricular hypertrophy [77]; it is noteworthy that mimicking a shift work schedule in mice similarly increases cardiac mass (and cardiomyocyte size) [21]. Collectively, these observational studies are consistent with the concept that challenging the myocardium with pro-hypertrophic stimuli (e.g., pressure, sympathetic tone) during the sleep phase augments cardiac growth. With this in mind, interventions should be tailored to specifically target these stimuli during the sleep phase for the reduction of cardiovascular disease risk. Evidence exists in support of this chronopharmacological strategy. For example, the large prospective MAPEC study reported that individuals taking at least one anti-hypertension medication prior to bedtime had a reduced risk of adverse cardiovascular events over an 8-year period [78]. Although the latter studies did not assess left ventricular mass, Martino et al. reported greater anti-hypertrophic efficacy of ACE inhibitors when given during the sleep period in an animal model of pressure overload [79]. It is noteworthy that some pharmacologic classes, such as β-blockers, may not be amenable to bedtime administration; β-blockers reduce melatonin levels and impair sleep [80]. Additional studies are therefore needed to determine the generalizability of anti-hypertrophic treatment strategies at bedtime.

## 7. Summary and Future Directions

In summary, through coordinated regulation of the multiple mechanisms that influence protein synthesis, the cardiomyocyte circadian clock primes the heart for growth specifically at the awake-to-sleep phase transition. Under non-stressed conditions, daily rhythms in cellular constituent turnover likely play an important role in the maintenance of cardiac function. However, in the presence of pro-hypertrophic stimuli and/or disease states, augmented cardiac growth at this time of day may facilitate adverse cardiac remodeling (and ultimately cardiac dysfunction). This raises the possibility that selectively decreasing hypertrophic stimuli at the awake-to-sleep transition through chronopharmacology has the potential to augment therapeutic effectiveness. However, several unanswered questions remain. With regards to mechanisms, whether one or more putative links between the cardiomyocyte circadian clock and cardiac growth highlighted thus far (Figure 3) play prominent roles is unclear, as is whether additional mediators exist. In the latter case, Sachan et al. have previously reported that calcineurin (a key regulator of cardiac hypertrophy) is circadian regulated in the heart, with increased activity at the awake-to-sleep phase transition [81]. Oxidative metabolism (which is temporally regulated by the cardiomyocyte circadian clock [11]) may also play a role; increased catabolism of amino acids during the first half of the active period has the potential to diminish the availability of essential building blocks for protein synthesis. It is also unclear whether the time of day favors the development of physiologic versus pathologic cardiac hypertrophy. Evidence supporting pathologic hypertrophy/remodeling at the awake-to-sleep phase transition includes observations that BCAA meal feeding at this time augments cardiac dysfunction in a murine model of cardiac disease (i.e., pressure overload) [22]. In contrast, relatively little is known regarding whether the time of day at which exercise is performed impacts the manner with which the heart remodels. Given that eating and exercise are considered common lifestyle behaviors that can be performed 24/7 in modern society, additional insight regarding their time-of-day-dependent impact on the human myocardium is needed. It is noteworthy that observational studies in humans strongly support the concept that distributing caloric intake towards the beginning of the awake period reduces cardiometabolic disease risk (although studies investigating left ventricular mass are lacking in humans) [82]. Consistent with this concept, preventing obese mice from consuming food during the sleep period for only 2 weeks is sufficient to reverse cardiac remodeling (including a normalization of cardiac mass and cardiomyocyte size), in the absence of changes in body weight or adiposity [83]. Moreover, recent animal-based studies indicate that exercise-induced changes in cardiac amino acid and nucleotide metabolites are dependent on the time of day; although such metabolites are important for growth, cardiac mass was not reported [84]. Future studies directly assessing the human myocardium following circadian-related interventions (i.e., time-of-day-specific caloric intake, exercise, and/or pharmacologic therapy) are undoubtedly needed.
